# Human Postural Control: Assessment of Two Alternative Interpretations of Center of Pressure Sample Entropy through a Principal Component Factorization of Whole-Body Kinematics

**DOI:** 10.3390/e20010030

**Published:** 2018-01-05

**Authors:** Thomas Haid, Peter Federolf

**Affiliations:** Department of Sport Science, University of Innsbruck, 6020 Innsbruck, Austria

**Keywords:** neuromuscular control, motor control, center of pressure COP, principal component analysis PCA, degrees of freedom, complexity, balance, neuroscience

## Abstract

Sample entropy (SaEn), calculated for center of pressure (COP) trajectories, is often distinct for compromised postural control, e.g., in Parkinson, stroke, or concussion patients, but the interpretation of COP-SaEn remains subject to debate. The purpose of this paper is to test the hypotheses that COP-SaEn is related (Hypothesis 1; H1) to the complexity of the postural movement structures, i.e., to the utilization and coordination of the mechanical degrees of freedom; or (Hypothesis 2; H2) to the irregularity of the individual postural movement strategies, i.e., to the neuromuscular control of these movements. Twenty-one healthy volunteers (age 26.4 ± 2.4; 10 females), equipped with 27 reflective markers, stood on a force plate and performed 2-min quiet stances. Principal movement strategies (PMs) were obtained from a principal component analysis (PCA) of the kinematic data. Then SaEn was calculated for the COP and PM time-series. H1 was tested by correlating COP-SaEn to the relative contribution of the PMs to the subject specific overall movement and H2 by correlating COP-SaEn and PM-SaEn. Both hypotheses were supported. This suggests that in a healthy population the COP-SaEn is linked to the complexity of the coordinative structure of postural movements, as well as to the irregularity of the neuromuscular control of specific movement components.

## 1. Introduction

Postural control of balancing tasks is a key aspect in many research areas, including sports training and balance capability assessment with clinical or sport oriented purpose. To quantify postural control past research has assessed the sample entropy (SaEn) of center of pressure (COP) trajectories, a variable that determines the irregularity of a time-series. Using SaEn, it has been shown that the postural control of patients suffering from concussion [1,2,3,4,5], autism spectrum disorders [6], vestibular schwannoma [7], multiple sclerosis [8,9], amongst others, is affected. Also, aging [10,11], overweight [12], and additional task difficulties [13] have been identified as factors that affect postural control.

How to explain differences in SaEn is still subject to debate. A popular way of interpreting higher SaEn values (high irregularity) of COP time-series is that they occur when the postural control system controls the movements in a more variable [8,14] or adaptable [15] way. The minimum intervention principle of optimal feedback control [16] suggests that movement variability and adaptability depend on the system’s necessity to control the degrees of freedom of the movement using various movement strategies. It remains unclear, however, if the irregularity originates from the coordination of different movement strategies (the “mechanical” degrees of freedom) or the irregularity of the neuromuscular control of specific movement strategies (the “neuromuscular” degrees of freedom).

Since COP time-series are calculated from the acting ground reaction forces [17,18] they do not possess the information about the exact body positioning. To identify the type of degrees of freedom that might be responsible for COP irregularity this study builds upon the comparison of COP data to kinematic data of participants performing a quiet stance [19]. The kinematic data is further analyzed via a principal component analysis (PCA) technique that allows for the factorization of movements into partial whole-body motions, called principal movements (PM) [20,21,22]. Each of the PMs determines one type of motion, i.e., one balancing strategy. The contribution of each of these PMs to the overall motion can be described by subject specific eigenvalues. The lower the contributions of lower-order PMs, the more different strategies are of importance to explain the overall movement. Following, if the mechanical degrees of freedom are responsible for the COP entropy, then (Hypothesis 1; H1) the COP entropy/irregularity should correlate inversely with lower-order subject specific eigenvalues. However, if the COP irregularity is linked to the neuromuscular control of movement strategies, then (Hypothesis 2; H2) the COP entropy should correlate with the entropy of those PMs that contribute most to the overall body displacement.

The aim of the current study is to assess the two interpretations of COP entropy by testing the two predictions H1 and H2 in COP and kinematic data, as obtained from healthy volunteers performing a bipedal stance.

## 2. Materials and Methods

### 2.1. Participants

The current paper analyzes data from a previous study in which twenty-one volunteers (11 males, 10 females, age 26.4 ± 2.4, weight 71 ± 10 (mean ± standard deviation)) were recruited. All of the participants had no recent injury or other balance affecting condition and were in good self-reported general health. The study protocol was approved by the Norwegian Regional Ethical Committee. The participants provided written informed consent prior to their participation.

### 2.2. Measurement Procedures

Before the measurement, volunteers were standing in front of the force plate. With the start of the measurement, they were instructed to step onto the force plate into a comfortable, hip-wide, bipedal stance with their hands on their hips and to maintain this position for 2 min, until they were signaled that the measurement had finished. Participants were asked to avoid any movements that were not required for postural control during their trial, such as, for example, scratching or turning the head. They were not specifically instructed to “stand as still as possible”, since we wanted to observe a relaxed stance rather than a stance with volunteers being concentrated on the avoidance of movements.

The volunteers were equipped with 27 reflective markers that were placed on the following anatomical landmarks: 1st metatarsophalangeal joint, posterior on the calcaneus, lateral malleoli, tibial shaft, lateral femoral condyles, thigh, trochanter major, crista iliaca, dorsal side of the wrist joint, lateral epicondyle, bilaterally on the acromion, manubrium, C7, and head (three markers on a custom-build adjustable helmet). The positions of these markers in a global (laboratory) reference system were captured at 300 Hz using a Qualisys motion tracing system consisting of 10 Oqus-400 cameras (Qualisys, Gothenburg, Sweden). Ground reaction forces were recorded at 1500 Hz using an AMTI—Optima force plate (AMTI, Watertown, MA, USA). The data-streams of the force plate and the cameras were synchronized on a computer running the Qualisys Track Manager software (Qualisys, Gothenburg, Sweden) and the three-dimensional (3D) positions of the markers, as well as the COP-positions were computed.

### 2.3. Data Analysis—COP Data Pre-Processing and Principal Component Analysis (PCA)

All further data analysis steps were conducted in Matlab R2015a (The MathWorks Inc., Natick, MA, USA). Both the first 20 and the last 20 s of each trial were omitted in the analysis to avoid instabilities due to stepping onto or off the plate or due to volunteers showing signs of impatience. The COP data was down-sampled to 300 Hz to resemble the kinematic data in terms of sample rate. Next, the COP-data was centered and represented in anteroposterior (COP*_AP_*) and mediolateral direction (COP*_ML_*).

**Figure 1:**
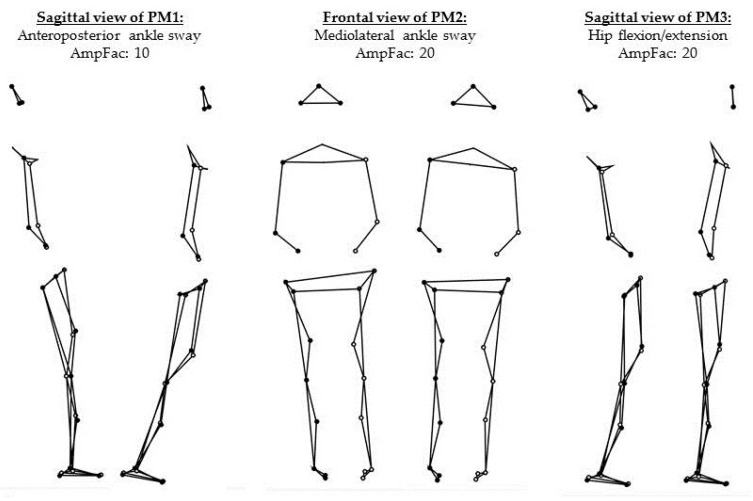
Visualization of the first three principal movement components (PM_1_–PM_3_) through the two positions with maximum deviation from the mean posture. For the visualization, the movement amplitude was further amplified with amplification factors (AmpFac).

The kinematic data was first normalized, by centering each data set of each participant separately and dividing by the participant’s height to reduce anthropometric differences. Then, gender specific relative weights were assigned to each marker [19,23]. As a next step, the normalized data sets of all the subjects were concatenated vertically into one input matrix for the PCA. Each row of the input-matrix represents the normalized posture of a volunteer at a given time-point [20,21,24,25]. Hence, the PCA was conducted in the 81-dimensional vector space of normalized posture vectors (27 markers with *x*, *y*, *z* component define an 81-dimensional posture vector). Performing the PCA determines a new orthonormal basis in this vector space with new basis vectors, called eigenvectors or principal component vectors (PC*_k_*, with *k* denoting the order of the eigenvectors). The PC-vectors are oriented in the direction of the largest variance in posture space. When applying PCA to kinematic data, each eigenvector PC*_k_* describes one linear pattern of motion (Figure 1) and thus represents a whole-body movement component. It has been suggested [19] to refer to these movement components as “principal movements” (PM*_k_*). The PCA also yields scores (PP*_k_*(*t*)) and eigenvalues (EV*_k_*). The scores are obtained by projecting the original data onto the new basis vectors, i.e., a basis transformation, and represent the posture vectors in the new basis. In accordance with previous publications [19], we call them principal positions (PP(*t*)), since they represent positions in posture space. The eigenvalues (EV*_k_*) quantify the overall contribution of each PM*_k_* to the overall variance that is produced by all of the subjects [20,21,24,25]. Since the EVs are not subject specific, it was proposed to calculate “relative variances” (rVAR) from the scores [26] that correspond to subject-specific relative EV*_k_*. However, both the rVARs and the EVs are proportional to the variance, i.e., to the square of the amplitude of postural movements. In the current study, we were interested in obtaining a variable that scales as the original data, and therefore we calculated the standard deviation (STD) of each subject specific PP*_k_*(*t*). Furthermore, the sum of all standard deviations of a volunteer’s PP*_k_*(*t*) can be interpreted as a measure of total amount of postural sway. Hence, to obtain the subject specific contribution of each PP*_k_*(*t*) to the postural movements of the respective subject, we calculated the relative standard deviation rSTD*_k_*, analogous to the relative variances [26], by calculating the percentage of contribution of each PP*_k_*(*t*)-STD to the total amount of postural sway. 

A leave-one-out cross-validation was performed to test the ability of the PCA-basis to represent the data. The first three PCs proved robust to subject changes and explained around 92.28% of the total variance. Each of the higher components contributed less than 2%. Therefore, the analysis in the current paper focuses on the first three PMs.

### 2.4. Calculation of Sample Entropy

Sample entropy (SaEn) was computed for the time-series of COP*_AP_*(*t*), COP *_COPML_*(*t*), and PP*_k_*(*t*) with *k* = 1,2,3 for the data of all volunteers. The SaEn calculation algorithm requires the specification of three computation parameters: embedding dimension *m*, tolerance *r*, and time-scale *τ*. In the current study, the parameters were set to *m* = 2 and *r* = 0.2·STD, which are typical choices for calculating COP-SaEn [27]. The selection of the time-lag was set to *τ* = 30 = 30/300 s = 100 ms since this time-delay is meaningful from a physiological point of view [28]. A sensitivity analysis (“SensitivityAnalysis_ComputationParameters.pdf”), attached to the current paper as supplementary materials, demonstrated that the ranking of the SaEn values, and, more importantly, the significance of the statistical evaluation, did not change for a wide range of these parameters. The parameter choice is therefore not crucial for the statistical significance or the interpretation of the results.

### 2.5. Statistics

Kolmogorov—Smirnov tests suggested a normal distribution in all the variables among the volunteers of this study. Therefore, Pearson’s correlations could be calculated.

Hypothesis H1 was tested by calculating the correlation between COP*_AP/ML_*-SaEn and rSTD_1,2,3_. A subject’s postural sway becomes more complex when higher-order movement components contribute more to overall movements, i.e., when the rSTD of the lower-order components decrease. Hence, a negative correlation was expected. The second Hypothesis H2 was tested by calculating the correlation between COP*_AP/ML_*-SaEn and PP_1,2,3_-SaEn. In this case, a positive correlation was expected, associating higher COP irregularity with higher irregularity of the control of the respective PP.

## 3. Results

A description of the movement strategy represented by each of the first three PMs can be found in Table 1. In addition, a graphical representation of these PMs can be found in Figure 1, as well as in the supplementary movie file “Visualization PM1-PM3.gif”. As an example, the movement strategy realization of the first subject can also be found in the supplementary materials (“Subject 1 PM1-PM3.gif”). The first two PMs resembled pure ankle strategies, whereas the third one can be interpreted as a hip strategy.

All of the correlations are summarized in Table 2. We found that COP*_AP_*-SaEn correlated specifically with PP_1_-SaEn and correlated negatively with rSTD_1_, but not with higher order PP*_k_* or rSTD*_k_*. COP*_ML_*-SaEn correlated with PP_2_-SaEn and correlated negatively with rSTD_2_. Hence, the hypothesized correlations were observed, and were specific to the postural movement component that represented a motion in the same direction as the COP-component: PM_1_ is an anteroposterior motion, while PM_2_ is a mediolateral motion. The PM_3_-related variables did not show any significant correlations with the COP entropies.

## 4. Discussion

### 4.1. Main Results

The current study tested two hypotheses, first, that the COP irregularity correlates with the amount of different movement strategies that contribute to the whole postural balance movements, and, second, that COP irregularity correlates with irregularity found in one-dimensional movement components, which would suggest that COP irregularity is influenced by the irregularity of the neuromuscular control signals that influence the corresponding direction the most. Both hypotheses were supported. 

The first hypothesis suggested that the relative sway (rSTD) in the PMs that are the main contributors to one of the COP-directions should show a negative correlation with the SaEn of the respective COP trajectory. When a lower rSTD is observed in one of the main contributing PMs then the other, higher-order movement components are more active and have a higher relative contribution to the overall movement. This means, in a sense, that the body sway consists of more coordinated movement strategies and the control system (voluntarily or involuntarily) utilizes more (combinations of) mechanical degrees of freedom. Since a balance movement that exhibits a more irregular COP displacement can be linked to more movement components that have to be controlled simultaneously, such a movement is more complex in terms of movement strategies to coordinate [14].

The second hypothesis was tested by correlating the SaEn of the COP time-series with the SaEn of the three main contributing PP*_k_*(*t*). Specifically, high irregularity in the anteroposterior and mediolateral direction of the COP-trajectory correlated with high irregularity in the anteroposterior and mediolateral ankle strategies PM_1_ and PM_2_, which are the main contributors to the respective center of mass displacement in the respective directions. This suggests that the irregularity of the COP trajectories is also linked to the neuromuscular control of individual postural movement components. Such irregularity might arise, for example, from the motor unit firing variability [29,30] and the irregularity that it produces in the activation of the muscles [31,32] that enable and control the specific movement component. 

In the framework of time-series analysis, a signal can be considered more complex if the signal contains more correlations and has higher SaEn values across multiple time-scales [8,33,34,35]. The fact that the SaEn values do not change their ranks notably when computing them over various timescales suggests that a more complex main contributing PP*_k_*(*t*) is linked to a more complex respective COP trajectory.

The results suggest that in our data the nonlinear dynamics of the PMs that relate to SaEn are preserved in the COP to some extent. Therefore, it should be possible to investigate the nonlinear dynamics of PMs in a similar fashion as the nonlinear dynamics of COP time-series. Future research should focus on the correlation of these dynamics in more challenging tasks or when the neuromuscular control system is impaired. If the origin of the COP complexity lies in the neuromuscular control of specific components, as proposed in this paper, the sample entropy of PP(*t*) should have the same capability to discriminate pathological groups as COP-trajectories with the advantage of knowing details about the exact types of movement the dynamics originate from.

### 4.2. Limitations

The neglected PMs explained almost 13% of the overall variance. Nevertheless, only three dimensions were used in this study, because the cross-validation showed that higher components were not useful to represent the group as a whole. Although it seems likely that the main dynamics were captured, the higher components could display additional interesting (subject specific) dynamics that were neglected in this study.

## 5. Conclusions and Outlook

This paper found that in healthy young adults the COP irregularity is linked to both the mechanical complexity of the postural movements and the irregularity of the neuromuscular control of specific movement strategies. We speculate that a similar analysis, as presented in the current study, applied to groups with specific clinical conditions might be able to clarify if these specific patients have difficulty with coordinating the mechanical degrees of freedom (mechanical complexity) or if they rather show changes in irregularity of the neuromuscular signaling.

## Figures and Tables

**Table 1:** Description of principal movement components PM*_k_*. Statistically significant correlations between center of pressure (COP)-sample entropy (SaEn) and Principal movement strategies (PM) variables are symbolized by *.

*k*	EV (Eigenvalues) (%)	Correlation	Main Strategy (Directions)	Specifications/Additional Features
1	77.77	*	Ankle sway (anterior/posterior)	No visible motions in the rest of the body.
2	9.71	*	Ankle sway (medial/lateral)	No visible motions in the rest of the body.
3	4.73		Hip strategy (flexion/extension)	Resulting upper body sway (posterior/anterior) visible.

**Table 2:** Pearson’s correlation coefficients *r_corr_* between the variables relative sway (rSTD) and SaEn of the PP*_k_* (relative standard deviation and sample entropy of principal positions) and COP*_AP/ML_*-SaEn (sample entropy of the center of pressure in AP and ML direction). SaEns were computed with parameters embedded dimension *m* = 2, tolerance *r* = 0.2·STD and time-lag *τ* = 30 = 100 ms.

	rSTD_1_		rSTD_2_		rSTD_3_		SaEn (PP_1_)		SaEn (PP_2_)		SaEn (PP_3_)	
	*p*	*r_corr_*	*p*	*r_corr_*	*p*	*r_corr_*	*p*	*r_corr_*	*p*	*r_corr_*	*p*	*r_corr_*
COP*_AP_*-SaEn	0.002	−0.633	0.705	0.088	0.261	0.257	<0.001	0.717	0.390	0.198	0.887	0.033
COP*_ML_*-SaEn	0.150	0.326	<0.001	−0.753	0.565	0.133	0.384	0.200	<0.001	0.893	0.730	0.080

## Supplementary Materials

The supplementary materials are available online at www.mdpi.com/1099-4300/20/1/30/s1. The PM visualizing files and the sensitivity analysis were uploaded as supplementary materials: Sensitivity analysis: “SensitivityAnalysis_ComputationParameters.pdf”, Figure S1: “PM1-PM3.jpg”, Video S1: “Subject 1-PM1-PM3.gif” and Video S2: “Visualization PM1-PM3.gif”. The analyzed data with its description were also uploaded as supplementary materials: COP-data “COPdata.mat”, kinematic-data “KinematicData.mat”, Data information: “DataInfo.txt” and marker number visualization “MarkersNumbered.jpg”.
