# Supplementary material for: Human Postural Control: Assessment of Two Alternative Interpretations of Center of Pressure Sample Entropy through a Principal Component Factorization of Whole-Body Kinematics
Source: Entropy (Basel). 2018 Jan 5;20(1):30. doi: 10.3390/e20010030 (PMC7512231; doi:10.3390/e20010030)

Sensitivity analysis for SaEn

The calculation algorithms for sample entropy (SaEn) utilize two parameters, tolerance “r” and time lag “tau”. The aim of this sensitivity study was to characterize how the SaEn result depends on these input parameters. Our input data had a sampling frequency of 300 Hz. In literature the standard tolerance level is $r = 0.2 \cdot STD$. Here we calculated SaEn for six tolerance levels (0.05, 0.1, 0.15, 0.2, 0.25, 0.3·STD).

Literature is not conclusive regarding the choice of the time lag tau. Tau depends on the sampling frequency and on the time scales that are of interest for specific study designs. A trend in literature is to compute multi-scale entropy by coarse graining time-series and comparing the irregularity over various time scales. However, a negative side effect is that coarse graining changes the distribution of the time series. In the current study we tested 61 time lags, starting from tau = 1 (corresponding to 1/300 seconds) in steps of 10 (tau = 10, 20, 30, …) till tau = 600 (corresponding to 2 seconds). In this analysis we adopted the reshape scale method introduced by Zandiyeh and von Tscharner (2013) *Physica A: Statistical Mechanics and its Applications*: 6265-6272.

Content

I. Tolerance level “r”
II. Time lag “tau”
III. Effect on statistical conclusions

I. Tolerance levels “r”

The tolerance level influences the value of sample entropy. Although the nominal value of the SaEn changes when the tolerance level is altered, the overall ranks of the SaEn values did not change notably between r = 0.05 and 0.3. Hence, r = 0.2 is a suitable choice for our study. The following graphs were computed exemplary with tau = 30=30/300=0.1 s = 100 ms.

1.1. $COP_{AP/ML}(t)$ - tolerance levels

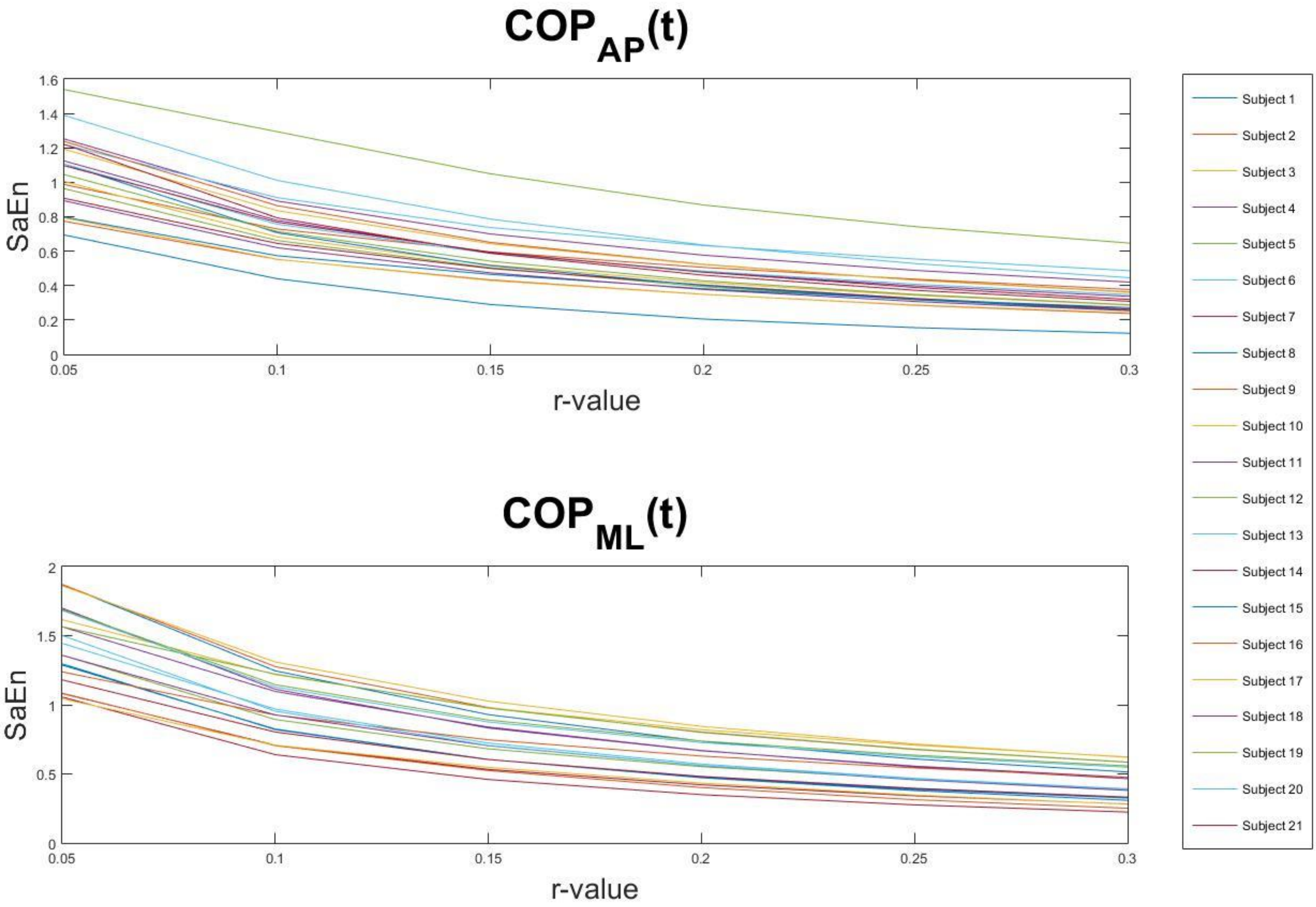

1.2. $PP_{1\text{-}3}(t)$ - tolerance levels:

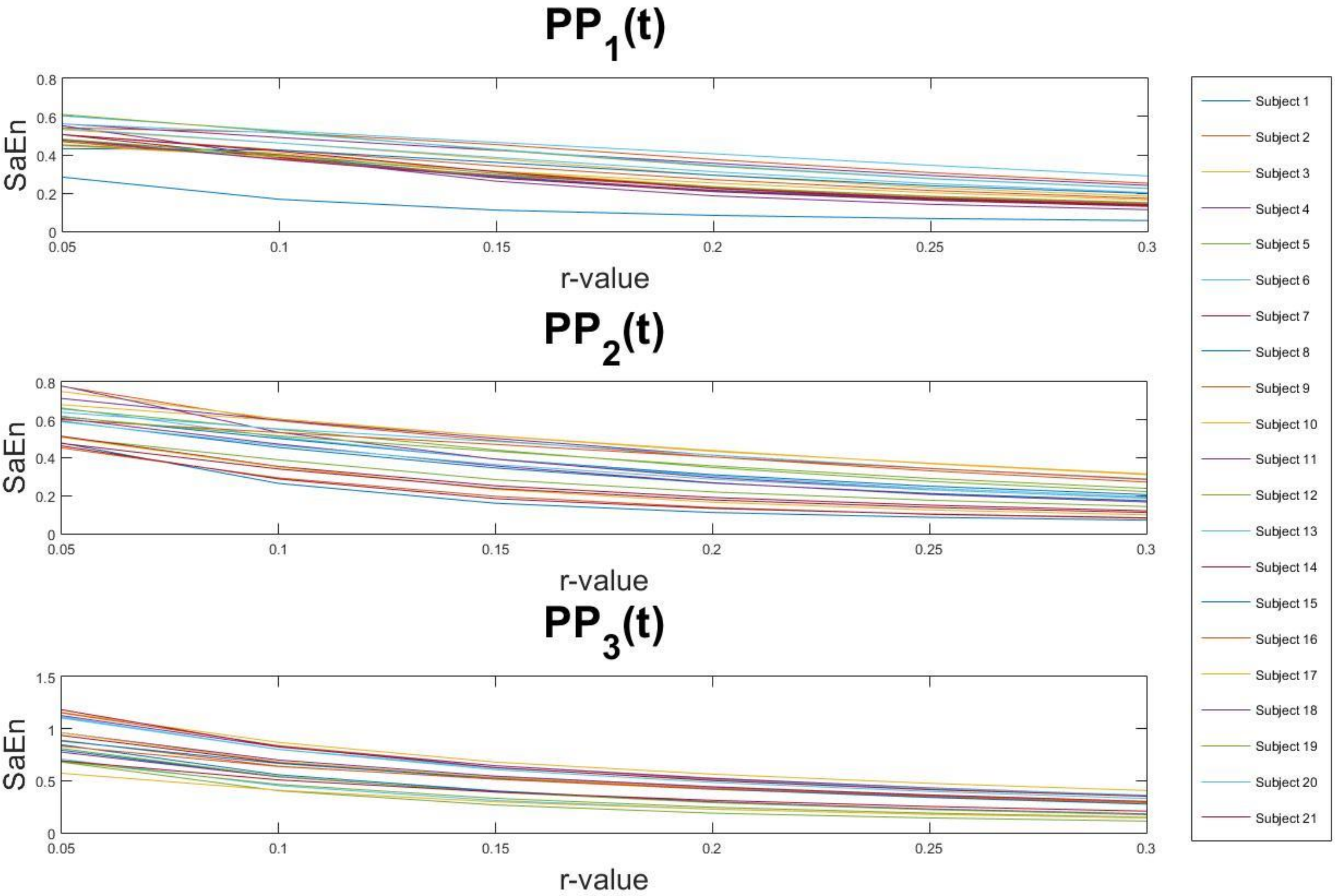

II. Time lag “tau”

A higher time lag results in a higher SaEn: at higher time scales the time-series of these postural control variables contain less self-similarity and are therefore less regular. The nominal values increase until tau ~ 200, before saturating. Therefore in the graphs and in the statistical assessment we focused on the range below tau = 300. However, despite the SaEn values changing their nominal values over different time-scales, the ranks remain approximately the same. In the current study we therefore chose a time lag of 30, which corresponds to 100 ms, since this time lag is well within the range and the time delay is meaningful from a physiological point of view (Kanekar et al., 2014). The following graphs were computed exemplary with r = 0.2.

2.1. $COP_{AP/ML}(t)$ - time lag

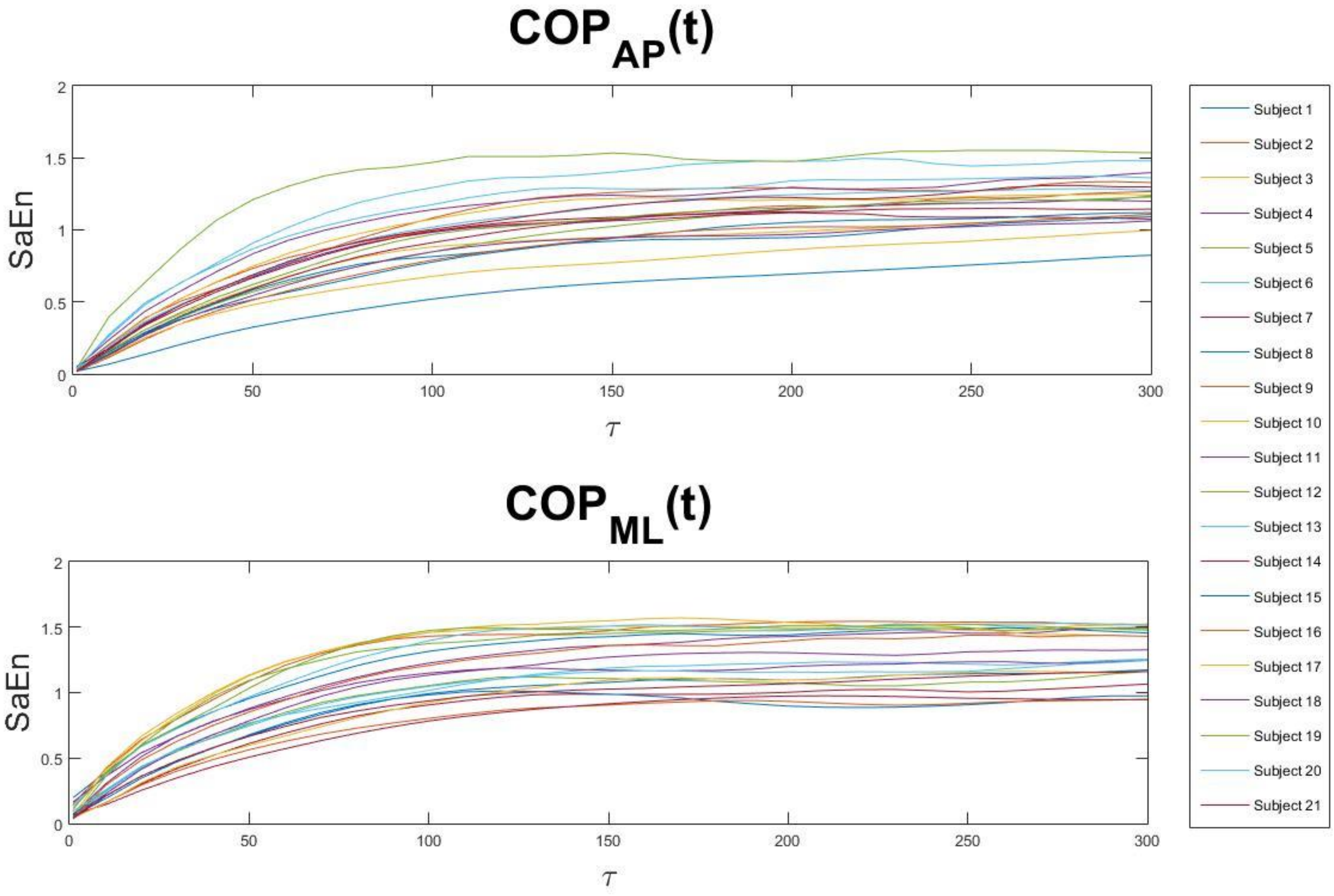

2.2. $PP_{1-3}(t)$ - time lag

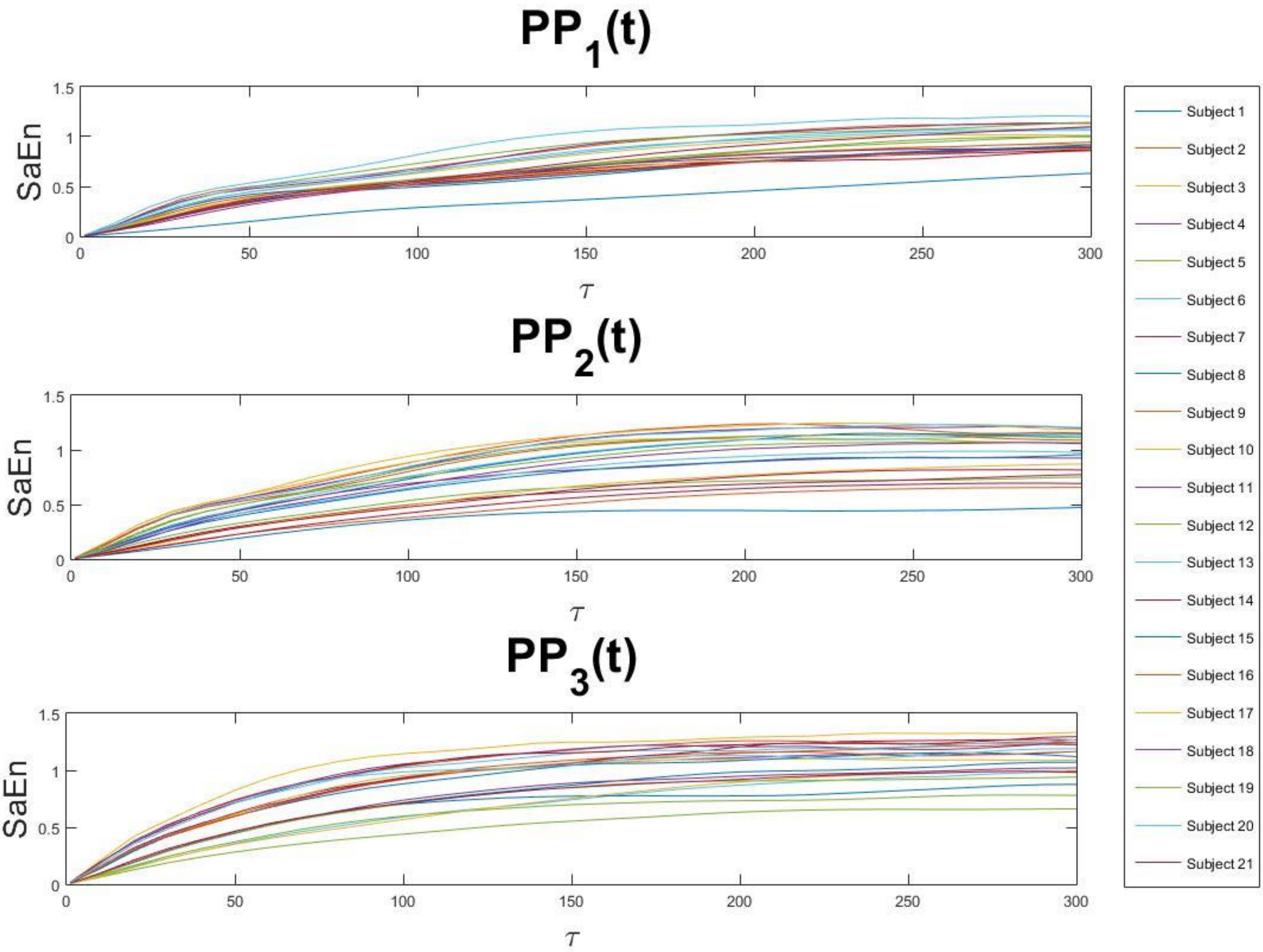

III. Effect on statistical conclusions

Finally, we compared the statistics over all parameter combinations. Due to the graphs in the previous section showing that the ranks of the entropy values do not change, we hypothesized that the statistics and the main results in our study are independent of the parameter selections. The following graphs show all p-values and all correlation coefficients for the computed parameter combinations. The statistical conclusions (significant or not significant) remained the same for almost all parameters. We observed that p-values of the statistically significant correlations remain very similar over a wide range of computation parameters, only the p-values of non-significant results fluctuated.

Similarly, significant correlation coefficients remained large independent of the computation parameters. The only exceptions were seen for tau = 1 for some r-tolerance levels and specific combinations of tolerance r = 0.05 with time lags > 100.

3.1 p-values of the SaEn(COP) - rSTD correlation

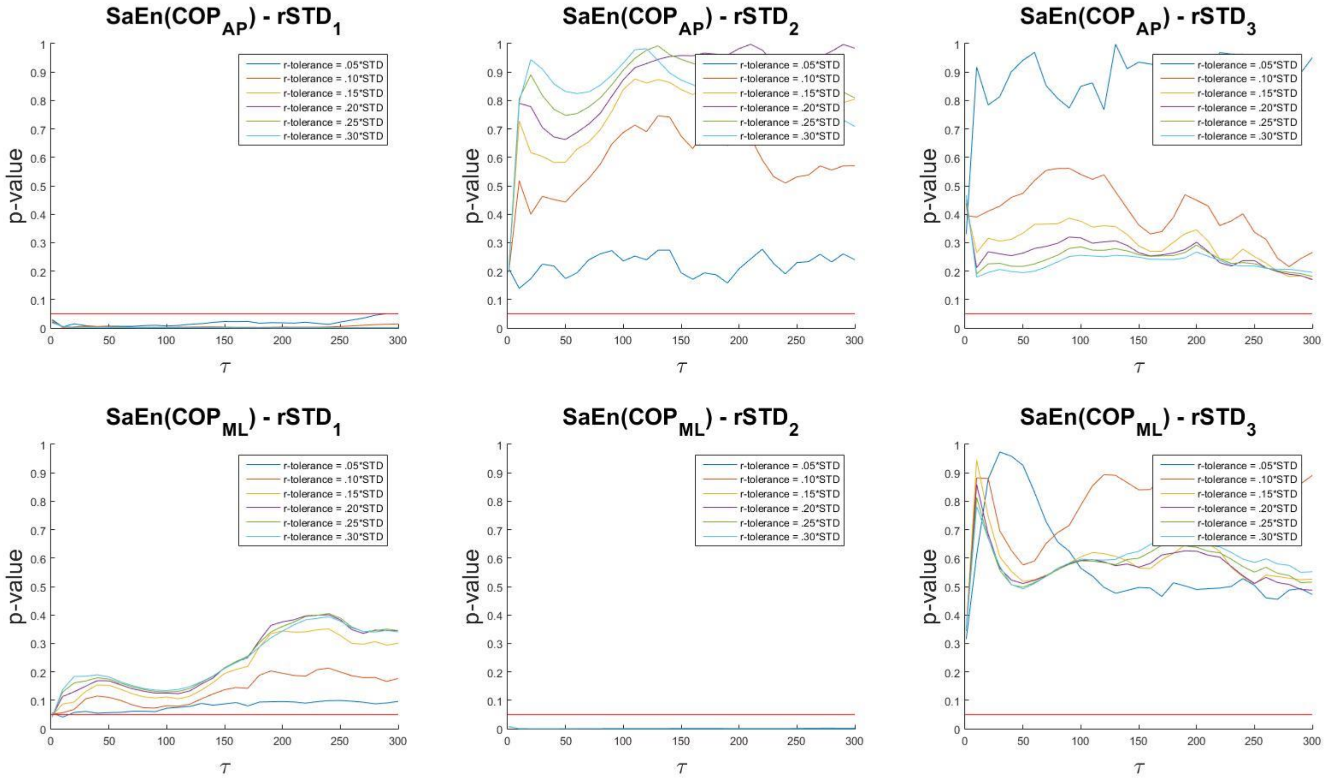

3.2 p-values of the SaEn(COP) - SaEn(PP) correlation

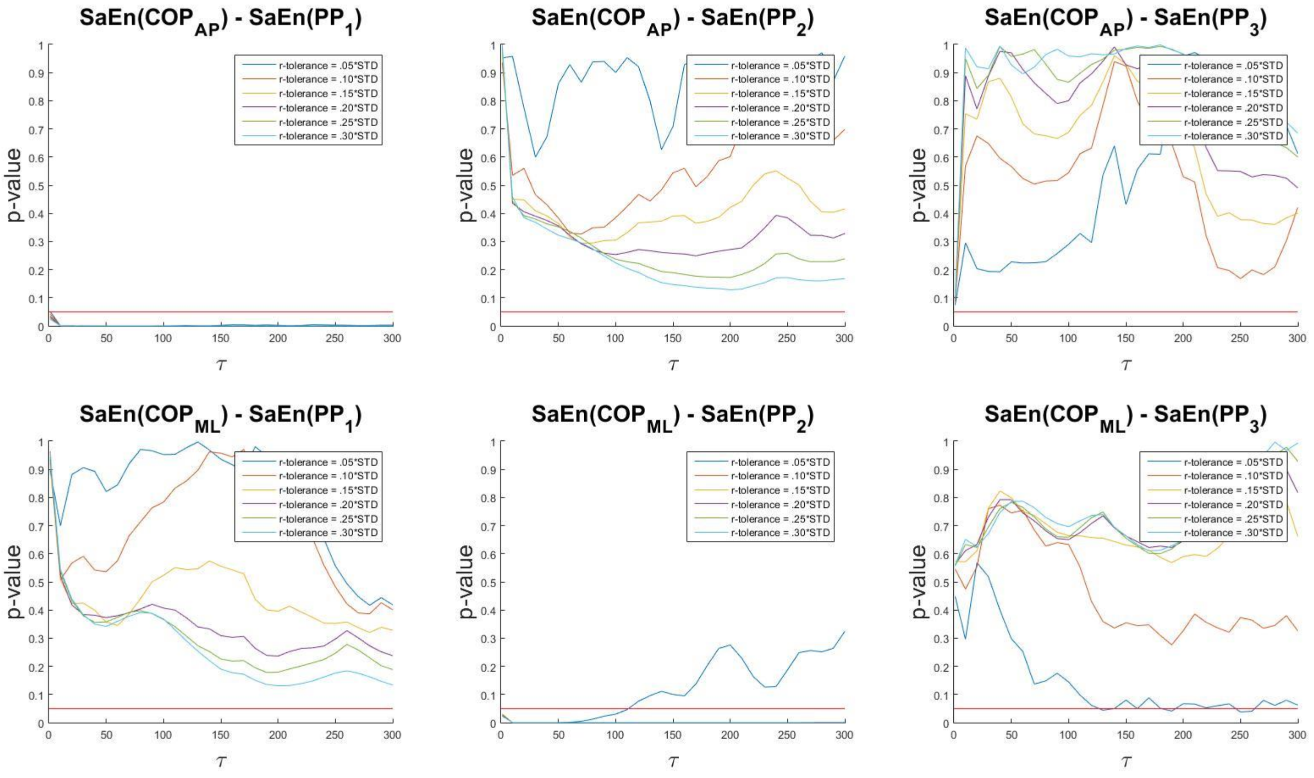

3.3 Correlation coefficients of the SaEn(COP) - rSTD correlation

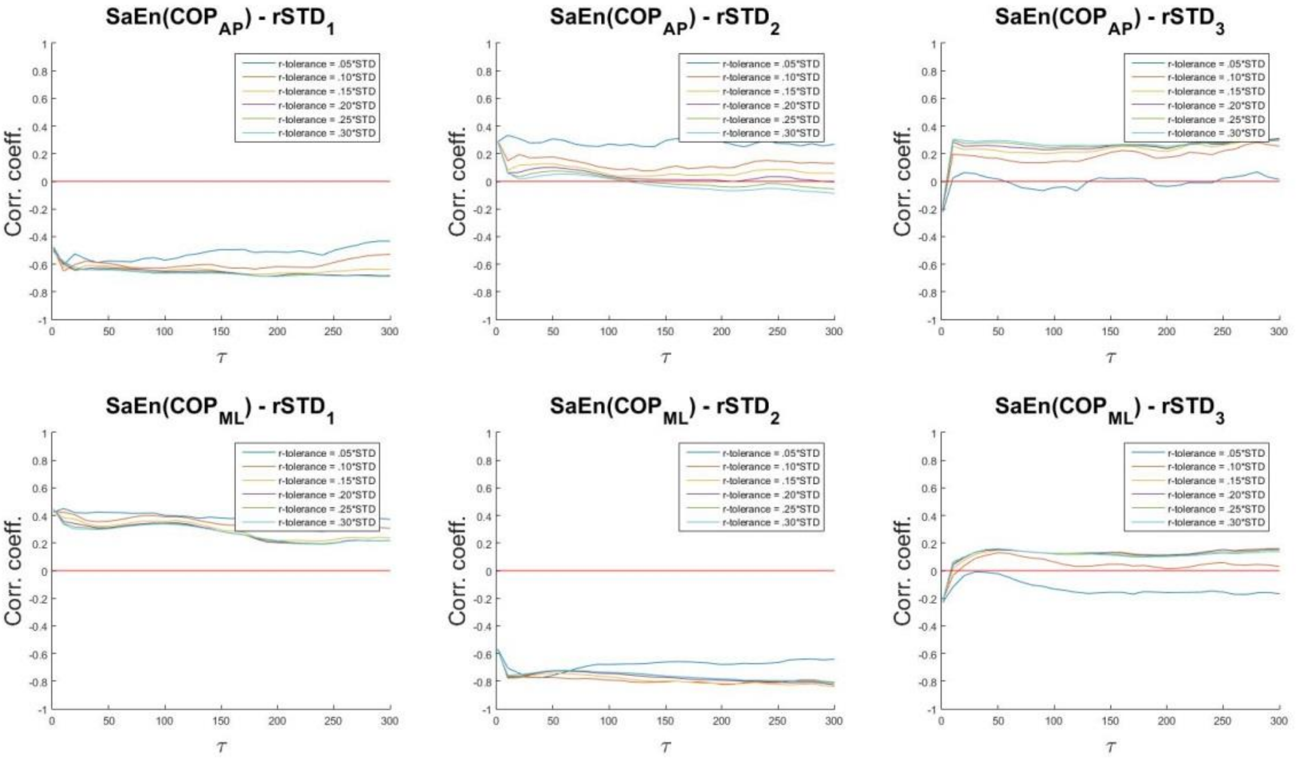

3.4 Correlation coefficients of the SaEn(COP) - SaEn(PP) correlation

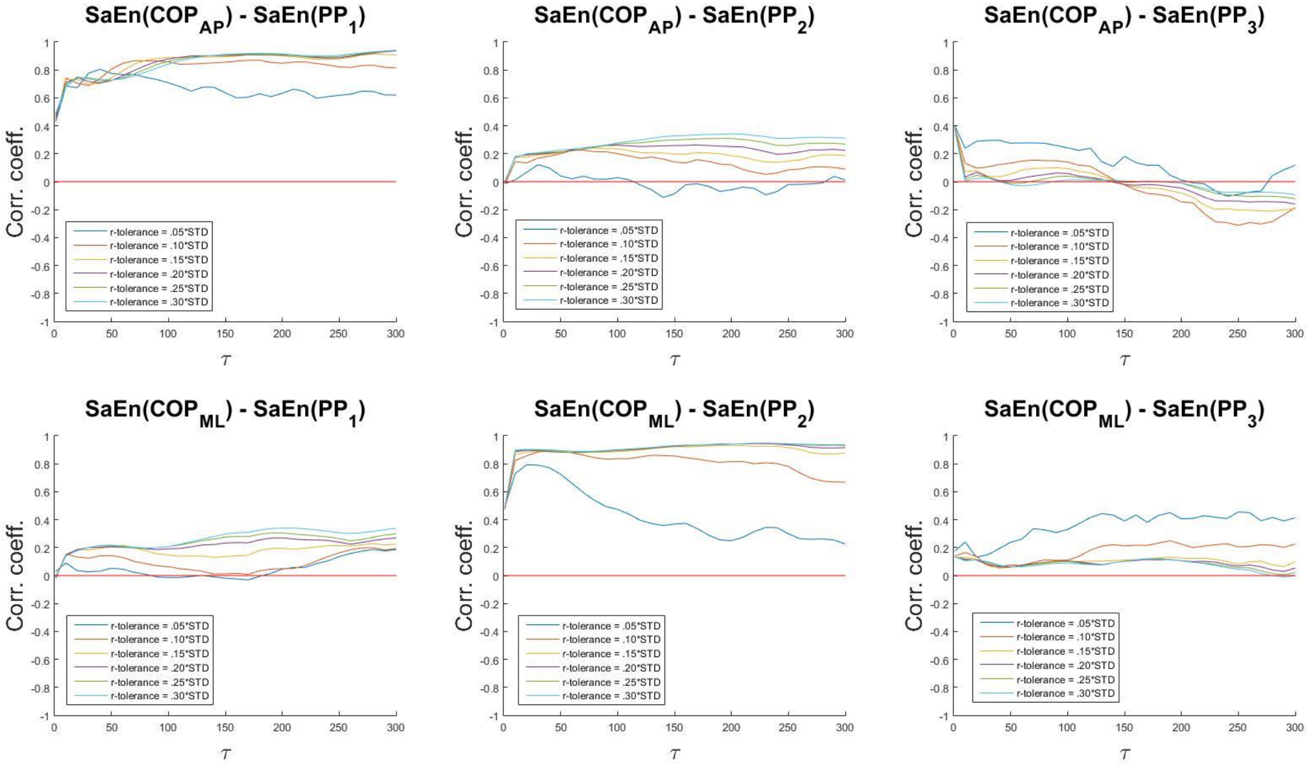

Supplement: Supplementary file 1 [file entropy-20-00030-s001.zip › SensitivityAnalysis_ComputationParameters.pdf]
