# Supplementary figures and images for: Human Postural Control: Assessment of Two Alternative Interpretations of Center of Pressure Sample Entropy through a Principal Component Factorization of Whole-Body Kinematics
Source: Entropy (Basel). 2018 Jan 5;20(1):30. doi: 10.3390/e20010030 (PMC7512231; doi:10.3390/e20010030)

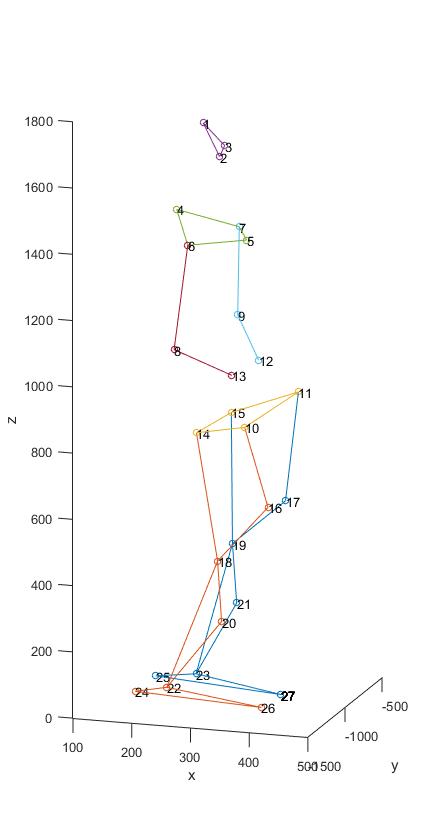

Supplement: Supplementary file 1 [file entropy-20-00030-s001.zip › MarkersNumbered.jpg]

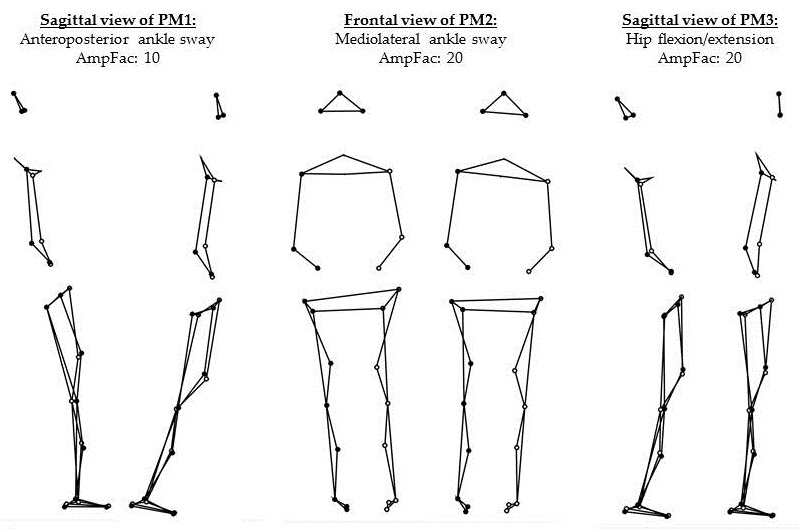

Supplement: Supplementary file 1 [file entropy-20-00030-s001.zip › PM1-3.jpg]

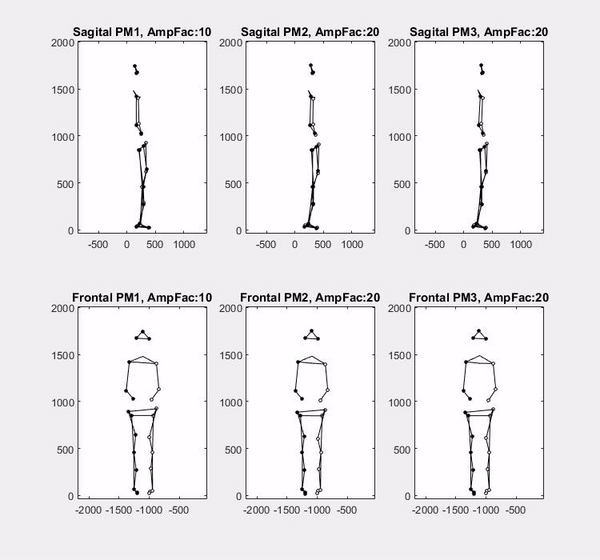

Supplement: Supplementary file 1 [file entropy-20-00030-s001.zip › Subject 1 - PM1-PM3.gif]

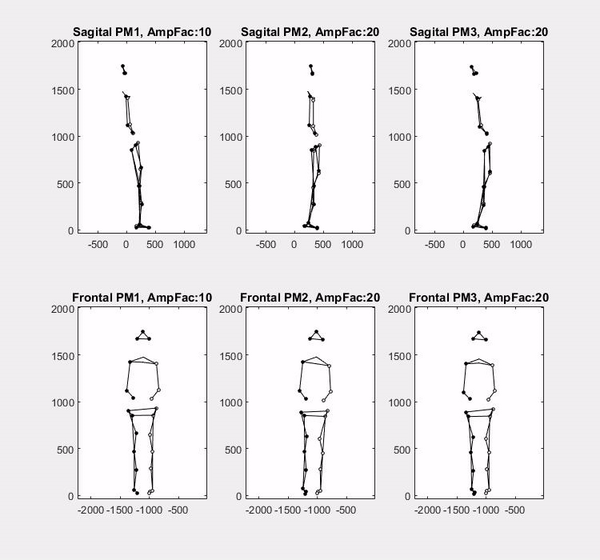

Supplement: Supplementary file 1 [file entropy-20-00030-s001.zip › Visualization PM1-PM3.gif]
